# Order type dataset analysis for fiducial markers

**DOI:** 10.1016/j.dib.2018.08.126

**Published:** 2018-08-31

**Authors:** Heriberto Cruz Hernández, Luis Gerardo de la Fraga

**Affiliations:** CINVESTAV, Computer Science Department, Av. IPN 2508, 07360 Mexico City, Mexico

## Abstract

Order Type (OT) describes a point set avoiding the use of metric information. We show that OT is a descriptor which is invariant to Euclidean geometric transformations, change of scale and perspective projection. In this paper we provide the data related to the application of Order Type with sets of 5, 6, 7, and 8 points to build fiducial markers. The OT is represented through a λ-matrix. We provide the set of points which are suitable to solve directly the point matching, because these have a unique associated λ-matrix. We provide maximal perturbation data for all set of points, maximal perturbation is the radius of the circle, centered in each point in the set, inside which each point can be moved without changing its associated OT. Also we provide the scripts to validate the use of OT in fiducial markers.

**Specifications table**

**Table:** 

Subject area	*Computer Vision*
More specific subject area	*Augmented reality, Fiducial tags, Order Type*
Type of data	*Files with data in ASCII, program scripts in bash shell, and C source code.*
How data was acquired	*Original Order Type instances are provided in*[1]*. We change the sets with 5, 6, 7, and 8 points to ASCII format. All the other data is generated with the provided scripts and programs.*
Data format	*Numbers and labels in ASCII separated by spaces.*
Experimental factors	*Experimental setup along its parameters described in*[2]
Experimental features	*Several quantitative and exhaustive experiments that validate the results in*[2].
Data source location	*N/A*
Data accessibility	*All files described in this paper are publicly available in*[3].
Related research article	*The data provided in this paper is the result of the research article in*[2].

**Value of the data**

• All data is provided to help to searchers and students in this field.
• The data about the set of points which are suitable to solve directly the point matching, can help pattern recognition researchers to choose and identify which are suitable for their own purposes.
• The maximal perturbation data for all set of points can be used to select a specific set of points in applications which require certain precision.
• The provided scripts and programs can be modified with other parameters to generate new data.
• The present work reduces the time and effort for other researches to introduce themselves to the order type concept.
• The provided datasets could help to develop and design other Order Type applications.

## 1 Data

This data is the result of the research paper in [2]. File names, description and attributes of the data are presented in Table 1. We provide data related to the OT instances suitable for point matching, two more related to the test of OT for Computer Vision applications, and one with the results of the maximal perturbation analysis (the robustness of OT to noise) [2]. Additionally we provide data and scripts to generate ray-traced images for testing OT as an Order Type Tag. All the data, except OT instances, are generated using the scripts presented in Table 2.

**Table 1:** Dataset specification.

**Dataset Name**	OT instances
**Description**	Order Type instances from [4] in ASCII format.
**Path**	/Data/OrderTypeInstances/
**Files**	C5.txt, C6.txt, C7.txt, and C8.txt.
	Cn.txt, where n indicates the point sets cardinality.
**Format**	• The number in each filename corresponds to the cardinality of the point sets inside. • One point set instance per line, each coordinate space separated, e.g., $x_{1}^{m}y_{1}^{m}x_{2}^{m}y_{2}^{m}x_{3}^{m}y_{3}^{m}\cdots x_{n}^{m}y_{n}^{m}$, where n referrers to the n-th point of the m-th point set instance.
**Dataset Name**	λ-matrices for point sets suitable for point matching
**Description**	Minimal λ-matrices for each point set instance, as well as verification of unique minimal λ-matrix. The λ-matrices correspond to the minimal lexicographical from the canonical orderings.
**Path**	/Data/PointMatchingSuitableOrderTypeInstances/
**Files**	E5.txt, E6.txt, E7.txt, and E8.txt.
	En.txt, where n indicate the point sets cardinality.
**Format**	• The l-th line of the file correspond to the λ-matrix of the point set at the corresponding l-th line of the Cn.txt files. • λ-matrix given as a vector with length n(n−1). Matrix diagonal elements of matrix are removed (these have no values). • Lines with a double λ-matrix indicate that there is not a unique minimal λ-matrix.
**Dataset Name**	Order Type instances Maximal Perturbation
**Descriptor**	Maximal Perturbation for each point set instance. It corresponds to the maximal noise that can be added to each point within the set instance without changing the associated Order Type.
**Path**	/Data/MaximalPerturbation/
**Files**	MP5.txt, MP6.txt, MP7.txt, and MP8.txt.
	MPn.txt, where n indicate the point sets cardinality.
**Format**	• Single real number per line. The l-th line of the MPn.txt file corresponds to the maximal perturbation of the point set at the l-th line of the Cn.txt files.
**Dataset Name**	Order type instances with specified maximal perturbation threshold
**Description**	Lists of Order Type instances with maximal perturbation values greater than a specified v threshold.
**Path**	/Data/MaximalPerturbation/MaximalPerturbationThreshold/v/
	for v=[0.5,1.0,…,8.5,9.0].
**Files**	MPlist5.txt, MPlist6.txt, MPlist7.txt, and MPlist8.txt.
	MPlistn.txt, where n indicates the point sets cardinality.
**Format**	Integer indices to the Cn.txt files. Contain only the line numbers of those point sets with maximal perturbation coefficients greater that the threshold v.
**Dataset Name**	Order Type rotation, tilt, and distance validation data
**Description**	Results of Order Type retrieval from images of Order Type instances in different conditions of rotation, tilt, and distance.
**Path**	/Data/SyntheticTest/TiltRotation/
**Files**	C7_tilt_0.txt, C7_tilt_20.txt, …, C7_tilt_80.txt, C8_tilt_0.txt, C8_tilt_10.txt, C8_tilt_20.txt, C8_tilt_30.txt, …, C8_tilt_80.txt, C7_distance.txt, and C8_distance.txt.
	Cn_tilt_θ.txt, where n indicates the point sets cardinality and θ the tilt angle.
**Format**	[l] [$\theta_{1}$] [$\theta_{2}$] [$t_{3}$][result]
	• l: index of the corresponding l-th point set of the corresponding Cn.txt file. • $\theta_{1}$: rotation angle from 0 to 350 in degrees. • $\theta_{2}$: tilt angle from 0 to 80 in degrees. • $t_{3}$: distance from camera center to the point set. • result: Success (in case that Order Type was correctly retrieved from the image) or Failure (in the contrary case).
**Dataset Name**	Order Type tags tilt test with ray-traced images
**Description**	Order Type tag seen in ray-traced images in different conditions of tilt and results of the Order Type retrieval.
**Path**	/Data/RayTracedImagesTest/
**Files**	./tag.png, image file used to generate the ray-traced images./Rotation/$\theta_{2}$.pgm, ray-traced images with the tag seen in different tilt angles with $\theta_{2}$ from -89 to 89./Rotation/result.txt, file with results of the experiment.
**Format**	[$\theta_{2}$] [result]
	• $\theta_{2}$: tilt angle in degrees. • result: Success (in case that Order Type was correctly retrieved from the image) or Failure (in the contrary case).
**Dataset Name**	Order Type tags distance test with ray-traced images
**Description**	Order Type tag seen in ray-traced images in different conditions of distance and results of the Order Type retrieval.
**Path**	/Data/RayTracedImagesTest/
**Files**	./Distance/t.pgm, ray-traced images with the tag seen in different distances from 30 to 200 cm./Distance/result.txt, file with results of the experiment.
**Format**	[$t_{3}$] [result]
	• $t_{3}$: distance in centimeters the value of $t_{3}$+29 value correspond to the distance between the camera and the tag in centimeters. • result: Success (in case that Order Type was correctly retrieved from the image) or Failure (in the contrary case).

**Table 2:** Description of the provided scripts.

**Script file**	**Description**	**Related table or section**
01EFiles.sh	It computes the dataset “λ-matrices for point sets suitable for point matching”.	Table 3 in [2]
02MPFiles.sh	It computes the dataset “Order Type instances Maximal Perturbation”.	Table 7 in [2].
03MPvData.sh	It computes the dataset “Order type instances with specified maximal perturbation threshold”.	Table 7 in [2].
04MPvFiles.sh	It is used by 03MPvData.sh to compute the dataset “Order type instances with specified maximal perturbation threshold”.	Table 7 in [2].
05SyntheticTiltTestData.sh	It computes the data related to tilt in the dataset “Order Type rotation, tilt, and distance validation data”.	Table 5 in [2].
06SyntheticDistanceTestData.sh	It computes the data related to distance in the dataset “Order Type rotation, tilt, and distance validation data”.	Table 6 in [2].
07RaytracedDistanceTestData.sh	It computes the dataset “Order Type tags tilt test with ray-traced images”.	Section 5.5 in [2].
08RaytracedDistanceTestData.sh	It computes the dataset “Order Type tags distance test with ray-traced images”	Section 5.6 in [2].
/ComputesLambdaMatrix	Directory with the source files of the program that computes the λ-matrix from a set of points.	Table 3 in [2].
/ComputesMaximalPerturbation	Directory with the source code to compute the maximal perturbation value for a given set of points.	Table 7 in [2].
/OrderTypeTagReader	Directory with the source files of the program to detect the Order Type Tags from pgm images and extract its triangles vertices, i.e., the tag׳s set of points.	Section 5.5 and Section 5.6 in [2].

## 2 Experimental design

**Fig. 1:**
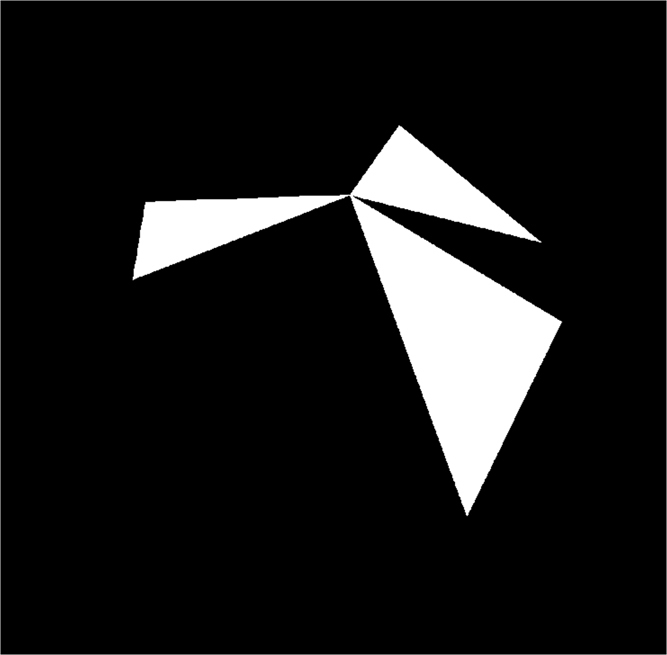
Order Type Tag used for the experiments with ray-raced images.

We performed different experiments to validate the proposed Order Type tags. First we transformed the point sets provided in [1] to ASCII format to obtain the Cn files. The data provided in this paper is the half of the data in Table 1 in [2], this corresponds to the set of points with cardinality within 5 to 8. For each set of points point sets we compute the λ-matrix and verify that a unique minimal λ-matrix exists. This allows us to identify the set of point that are suitable for solving directly the point matching problem. Later, we compute the maximal perturbation values and we find the set of points with a maximal perturbation value greater than a threshold v, with v from 0.5 to 9.0 in steps of 0.5. Later, we generated synthetic images with the camera pinhole model to verify that OT can be retrieved from the set of points in perspective. Additionally, we test the Order Type tags with ray-traced images. We generated scenes using the Order Type Tag in Fig. 1 with different conditions of tilt and distance and we verified the correct tag identification. The generated datasets are described in Table 1 and the scripts associated to each dataset are presented in Table 2. Details, of the validations, experiments, parameters and implementations are detailed in [2].
